# Exploring Neutrophil Extracellular Traps in Cardiovascular Pathologies: The Impact of Lipid Profiles, PAD4, and Radiation

**DOI:** 10.32604/biocell.2025.062789

**Published:** 2025-06-24

**Authors:** Siarhei A. Dabravolski, Michael I. Bukrinsky, Aleksandra S. Utkina, Alessio L. Ravani, Vasily N. Sukhorukov, Alexander N. Orekhov

**Affiliations:** 1 Department of Biotechnology Engineering, Braude Academic College of Engineering, Karmiel, 2161002, Israel; 2 School of Medicine and Health Sciences, The George Washington University, Washington, DC 20037, USA; 3 Department of Commodity Expertise and Customs Business, Plekhanov Russian University of Economics, Moscow, 115054, Russia; 4 Institute for Atherosclerosis Research, Moscow, 121609, Russia; 5 Institute of General Pathology and Pathophysiology, Moscow, 125315, Russia; 6 Institute of Human Morphology, Petrovsky Russian National Center of Surgery, Moscow, 119991, Russia; 7 Faculty of Biology and Biotechnology, National Research University Higher School of Economics, Moscow, 117418, Russia

**Keywords:** Neutrophil extracellular traps, atherosclerosis, myocardial infarction, citrullination, radiation, cardiovascular diseases

## Abstract

Neutrophil extracellular traps (NET) have emerged as critical players in the pathogenesis of atherosclerosis and other cardiovascular diseases (CVD). These web-like structures, composed of DNA, histones, and granule proteins released by neutrophils, contribute significantly to both inflammation and thrombosis. This manuscript offers a comprehensive review of the recent literature on the involvement of NET in atherosclerosis, highlighting their interactions with various pathophysiological processes and their potential as biomarkers for CVD. Notably, the impact of radiation on NET formation is explored, emphasising how oxidative stress and inflammatory responses drive NET release, contributing to plaque instability. The role of histones, particularly citrullinated histones, in endothelial dysfunction and plaque progression is discussed, highlighting their significance in the pathophysiology of atherosclerosis. Furthermore, the complex relationship between lipoproteins and NET formation is examined, with a focus on how elevated low-density lipoprotein (LDL) and decreased high-density lipoprotein (HDL) levels facilitate NET release, thus promoting vascular inflammation and plaque instability. The influence of cholesterol on NET formation is also explored, underscoring its contribution to plaque development and stability. The role of Peptidylarginine deiminase 4 (PAD4) in the regulation of NETosis is reviewed, with attention given to how PAD4-driven citrullination of histones affects atherosclerosis progression. Moreover, the manuscript examines the potential of NET components—such as double-stranded DNA, myeloperoxidase–DNA complexes, and citrullinated histone H3—as biomarkers for assessing disease severity and predicting adverse cardiovascular events, including ST-elevation myocardial infarction (STEMI) and stroke. Elevated levels of these biomarkers correlate with worse clinical outcomes, suggesting their utility in guiding therapeutic interventions. In contrast to the existing body of work, this review highlights the novelty of integrating recent findings on NET interactions with lipid metabolism, histone modifications, and PAD4 activity in the context of atherosclerosis. Overall, NET plays an integral role in the inflammatory and thrombotic processes underpinning atherosclerosis, and their components hold promise as both diagnostic markers and therapeutic targets in cardiovascular disease management.

## 1 Introduction

Atherosclerosis (AS) is a chronic inflammatory condition involving arterial wall changes, lipid deposition, and atheromatous plaque formation. It underlies cardiovascular diseases (CVD) such as coronary artery disease (CAD), peripheral artery disease, aneurysms, and myocardial infarction (MI). Collectively termed atherosclerotic cardiovascular diseases (ASCVD), these conditions cause approximately 17.9 million deaths annually, accounting for 32% of global mortality [1]. AS develops in three stages: initiation, progression, and advanced complications. Its progression is influenced by diabetes, obesity, dyslipidaemia, hypertension, hyperglycaemia, poor diet, inactivity, chronic stress, genetic predisposition, and environmental factors [2]. Key mechanisms include lipid accumulation, oxidative stress, and inflammation, resulting in plaque growth, foam cell accumulation, calcification, and necrotic core formation. Advanced plaques can obstruct blood flow, increasing the risk of rupture and thrombosis.

Low-density lipoprotein (LDL), known as “bad cholesterol”, is essential for lipid transport but strongly linked to AS and ASCVD risk [3,4]. LDL particles infiltrate the arterial intima during early atherogenesis, undergoing modifications such as oxidation, glycation, and carbamylation, whose effects on lesion development are poorly understood. These multiple modified LDL (mmLDL) forms promote inflammation, foam cell formation, and immune activation [5,6]. While oxidised LDL (oxLDL), a collective term used to describe quite different types of LDL modifications, has been extensively studied, its role in AS initiation remains uncertain [7]. Other modified forms, such as desialylated LDL, are emerging therapeutic targets [8]. The causal role of low-density lipoprotein cholesterol (LDL-C) in AS is well-established through pharmacological and epidemiological studies [9]. High-density lipoprotein cholesterol (HDL-C), or “good cholesterol,” is generally associated with reduced ASCVD risk. However, therapies to elevate HDL-C levels have not consistently improved outcomes [10]. Meanwhile, triglyceride-rich lipoproteins and lipoprotein(a) (Lp(a)) are recognised as significant contributors to AS [11,12].

Inflammation plays a key role in AS, closely linked to lipid accumulation. mmLDL aggregates in the arterial intima, inducing endothelial cells (EC) dysfunction and upregulating adhesion molecules like vascular cell adhesion molecule 1, intercellular adhesion molecule 1, and selectins, facilitating leukocyte adhesion. Activated EC, vascular smooth muscle cells, and leukocytes release inflammatory mediators that perpetuate local inflammation and immune responses [13,14]. Key inflammatory molecules, including tumour necrosis factor-alpha (TNF*α*), interleukins (IL-1*β*, IL-6), interferon-gamma, and C-reactive protein, serve as therapeutic targets and biomarkers of cardiovascular risk [15–18]. However, targeting inflammatory molecules to prevent atherosclerosis has proved challenging. Thus, the application of therapeutic monoclonal antibody targeting interleukin-1*β* in patients with previous myocardial infarction reduced the rate of recurrent cardiovascular events, while was associated with a higher incidence of fatal infection and sepsis [19].

Oxidative stress exacerbates endothelial dysfunction and AS progression. Excess reactive oxygen species (ROS) and reduced antioxidant defences amplify EC damage, lipid deposition, and inflammation [20,21]. ECs regulate vascular permeability and secrete molecules critical for anticoagulant and haemostatic functions. Lipid deposition and macrophage infiltration drive foam cell formation, triggering inflammatory cytokine release and processes like endothelial-to-mesenchymal transition. These changes impair endothelial function and accelerate plaque growth. Advanced plaques often feature vulnerable necrotic lipid cores capped by thin fibrous layers, prone to rupture and thrombosis [22,23].

Disrupted nitric oxide (NO) production further impairs vascular health. Under normal conditions, endothelial nitric oxide synthase (eNOS) regulates NO levels, maintaining vascular homeostasis. Pathological conditions suppress eNOS and elevate vasoconstrictors, promoting thrombosis and atherosclerosis [24,25].

As atherosclerosis advances, plaques accumulate more lipids and blood cells, further driving the formation of foam cells, which, eventually die by apoptosis and accumulate as apoptotic cells. Smooth muscle cells (SMC) transdifferentiation into foam cells also contributes to plaque growth [26]. Programmed cell death of smooth muscle cells and macrophages, combined with impaired efferocytosis (the clearance of apoptotic cells), leads to the development of a necrotic core within the plaque [27]. Additionally, disruptions in calcium metabolism within plaques initiate a mineralisation process akin to bone formation [28]. Localised, or “spotty” calcification often destabilises plaques, increasing their susceptibility to rupture and thrombosis, although more extensive calcification may stabilise plaques in some cases [23].

Plaque growth narrows the arterial lumen, increasing risks of ischaemia and angina pectoris [29]. Vulnerable plaques with large lipid cores and thin caps are prone to rupture, while stable plaques with thicker caps pose a lower immediate risk. Plaque erosion, a distinct mechanism involving less inflammation and lipid accumulation, also contributes to thrombosis but is less understood [30,31].

### NET in Atherosclerosis

**Figure 1:**
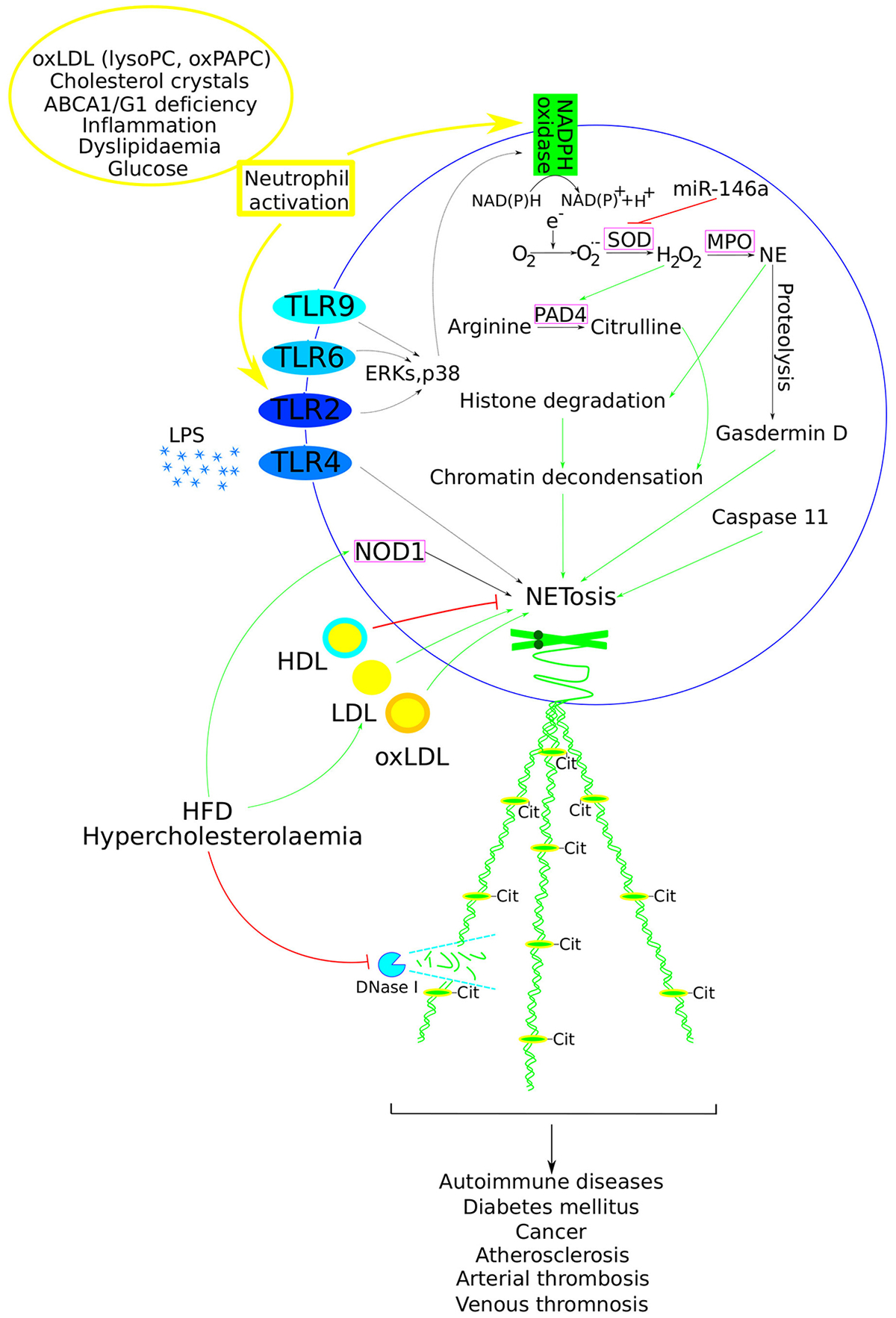
Critical events implicated in NET formation and its role in various pathophysiological conditions. Atherosclerosis risk factors can trigger NETosis within blood vessels (listed in the yellow circle). NADPH oxidase produces ROS, such as superoxide anion (O_2_^−^) and hydrogen peroxide (H_2_O_2_), which subsequently affect PAD4. PAD4 catalyses the deimination of arginine residues in histones, producing citrulline. The citrullination of histones leads to chromatin decondensation, a crucial step in NET formation. Additionally, MPO promotes NE release from the azurophilic granules in a ROS-dependent manner. NE is further involved in histone degradation, thus contributing to chromatin decondensation and NET formation. Simultaneously, NE mediates the proteolytic activation of the pyroptosis-associated protein gasdermin D, which, together with caspase-11, also mediates NETosis. TLRs are important activators of NET, acting through various signalling pathways (such as ERK and p38) (missing steps are depicted with dotted arrows), promoting NET formation. High levels of LDL and oxLDL, associated with HFD and hypercholesterolaemia, have been shown to promote NETosis, whereas HDL suppresses it. Moreover, hypercholesterolaemia has been shown to suppress DNase activity, thereby impairing NET clearance. NOD1 represents another NET-promoting mechanism activated by HFD. MiR-146a promotes NET formation by targeting SOD2, thereby enhancing ROS production. Finally, NET participates in the development of other diseases. Positive regulation is depicted with green arrows, while negative regulation is indicated with red blunt lines. Abbreviations: Nicotinamide adenine dinucleotide phosphate (NADPH); reactive oxygen species (ROS); peptidyl arginine deiminase 4 (PAD4); myeloperoxidase (MPO); neutrophil elastase (NE); toll-like receptors (TLRs); extracellular signal-regulated kinases (ERK); oxidised low-density lipoprotein (oxLDL); high-fat diet (HFD); high-density lipoproteins (HDL); superoxide dismutase (SOD); nucleotide-binding oligomerisation domain 1 (NOD1)

In recent years, the role of neutrophils in atherosclerosis has become better understood [32]. Thus, neutrophils promote plaque progression via myeloperoxidase (MPO)-mediated LDL oxidation, reduce plaque stability by secreting matrix metalloproteinases that degrade the extracellular matrix, cause endothelial damage by released reactive oxygen species, and contribute to plaque destabilisation by secreting NET [33]. NET are network-like structures released by activated neutrophils, originally identified as a unique immune defence mechanism distinct from the direct engulfment of pathogens by phagocytosis [34]. For example, among 24 identified NET-associated proteins, heterodimer calprotectin was recognised as the major NET-released antifungal component involved, in particular, in host defence against *Candida albicans* infection [35]. Another research deciphering the role of injury-associated proteases (such as thrombin and plasmin) on NETome has identified a total of 164 proteins, some of them, such as serine protease inhibitor (Serpin)-B1, lipocalin-2, complement C3, and antimicrobial proteins cathelicidin and S100A12, were not previously described to be present on NETs [36]. Furthermore, these structures contain chromatin, histones, granule proteins, MPO, and many other proteins (a total of 330) [37]. NET plays a dual role in the human body; while they provide a protective function in innate immunity [38], their excessive accumulation can become toxic to host cells, triggering autoimmunity and resulting in tissue damage [39]. NET has been implicated in tissue damage, inflammation [40], tumour development and metastasis [41,42], and various autoimmune conditions, including systemic lupus erythematosus [43] and rheumatoid arthritis [44] (Fig. 1). Recent research has shown that NET can promote the release of pro-inflammatory molecules, such as IL-8, further mediating inflammation and enhancing the inflammatory milieu [45,46].

Initially, Brinkmann et al. defined NET as web-like structures released by neutrophils to create a physical barrier that impedes bacterial spread [34]; however, subsequent research has characterised NET formation as a unique form of cell death termed NETosis [47]. The main components of NET include neutrophil elastase (NE), MPO, tissue protease G, and DNA, among others. NET formation occurs via two primary pathways: NADPH oxidase (Nox)-dependent and Nox-independent [48,49]. The translocation of MPO and NE to the nucleus is critical for NETosis, a process dependent on actin-myosin II cytoskeletal rearrangements [50,51]. Upon neutrophil activation, the immune-related GTPase family M protein activates signalling pathways involving p38, c-Jun N-terminal kinase, and mitogen-activated protein kinase 7, increases *cytosolic phospholipase A2* expression, and enhances levels of NE, MPO, and histone H3 [52] (Fig. 1). Gasdermin D, a protein associated with the pyroptosis, is proteolytically activated by NE and, along with caspase-11, mediates NETosis [53]. Peptidylarginine deiminase 4 (PAD4), a calcium (Ca^2+^)-dependent enzyme, plays a crucial role by citrullinating arginine residues. This citrullination leads to the rapid breakdown of the actin cytoskeleton within neutrophils, resulting in the shedding of plasma membrane microvesicles, disassembly of microtubules and vimentin, chromatin decondensation, and the release of DNA and intracellular proteins [54,55]. In basic research, lipopolysaccharide (LPS) and phorbol-12 myristate-13 acetate (PMA) are commonly used to induce NET, which typically appears a few hours post-stimulation [46,56].

Recent studies show that NET is implicated not only in infectious diseases but also in non-infectious conditions like atherosclerosis, which is driven by both inflammatory processes and dysregulation of lipid and glucose metabolism. Factors such as elevated cholesterol, oxidative stress, high glucose levels, various complement proteins, and pro-inflammatory mediators all contribute to the development of atherosclerosis [16,57–59]. Further in this review, we discuss the role of various factors (such as histones, PAD4, cholesterol, LDL/HDL, radiation, and miR-146a) in regulating NET formation and their contribution to inflammation, plaque development, and stability. We also examine the potential of NET as biomarkers in CVD, with a specific focus on their association with acute STEMI, stroke, and other cardiovascular events. While the role of MPO in atherosclerosis, particularly in its interactions with LDL and HDL, has been extensively addressed in several recent reviews [60–65], and the progress in therapeutic applications of Histone Deacetylases (HDAC) inhibitors (both selective and pan-HDAC inhibitors) in atherosclerosis has been thoroughly explored [66–71], this manuscript aims to bring new insights by integrating the latest findings in the field. These include novel aspects of NET regulation by cholesterol, histone modifications, PAD4, and radiation, alongside emerging evidence on miR-146a’s role. The aim of this review is to provide a comprehensive and updated perspective on how NETs contribute to atherosclerosis, beyond previously covered topics, and to highlight their potential as novel biomarkers and therapeutic targets in cardiovascular disease. Accordingly, we have excluded these well-established areas from the current review and redirect interested readers to the cited publications for more detailed discussions.

## 2 Histone Modifications in Neutrophil Extracellular Traps: Implications for Atherosclerosis and Vascular Pathology

Histones are significant components of NET, released into the extracellular matrix and citrullinated in a PAD4-dependent manner during NETosis. Histones (H1, H2, H3, and H4) play critical roles in regulating EC damage, inflammation, and cell death. For instance, stimulation of Human Umbilical Vein Endothelial Cells (HUVEC) with citrullinated H3 (citH3) disrupts the microvascular endothelial barrier by opening adherens junctions and reorganising the actin cytoskeleton, although it does not induce cell toxicity [72]. Additionally, histones bind to phospholipids in epithelial and EC membranes, increasing membrane permeability and calcium influx, which can lead to cell death and tissue damage [73]. Histone modifications, such as acetylated histone H4 (AcH4), are involved in promoting NET formation, with AcH4 enhancing both Nox-independent and Nox-dependent NETosis through chromatin decondensation mediated by ROS [74].

Although there is currently no evidence linking histone methylation to NETosis, histones exert various biological effects through Toll-like receptors, resulting in different forms of tissue damage or necrosis. Furthermore, as regulators of cell injury or necrosis, histones promote leukocyte recruitment to damaged tissues, enhance immune cell adhesion, and stimulate additional NET release, thus perpetuating a vicious cycle of inflammation [75].

In this context, oxLDL facilitated neutrophil recruitment, while both LDL and oxLDL increased NETosis (Fig. 1). LDL oxidation, aggregation, and foam cell formation were also elevated in the presence of NET. Citrullinated histones (CH–P and CH-M groups) did not affect LDL oxidation but accelerated LDL aggregation and foam cell formation at higher citrulline levels. These results suggest that both NET and NET-associated citrullinated histones released at the lesion site may act as pro-atherogenic factors [76].

Furthermore, neutrophil adhesion, NE and MPO levels, and citH3 in circulating neutrophils were increased in high-fat diet (HFD)–fed low-density lipoprotein receptor–null (*LDLR*^*−/−*^) mice compared to wild-type mice (Table 1). Interestingly, plasma from HFD-fed *LDLR*^*−/−*^ mice facilitated histone H3 citrullination. Further analysis identified C-X-C Motif Chemokine Ligand 1 (CXCL1) as the factor responsible for increased citH3 in the circulating neutrophils of *LDLR*^*−/−*^ mice. Stepwise application of CXCL1 and PAD4 inhibitors demonstrated that inhibition of citrullination in neutrophils reduced neutrophil adhesion to EC induced by CXCL1 stimulation. Finally, the application of the PPAR*α* agonist pemafibrate normalised the lipid profile, reduced neutrophil adhesion, CXCL1 levels in the blood, and citH3 levels in circulating neutrophils. This study collectively shows that dyslipidaemia can affect citH3 levels in neutrophils through CXCL1 upregulation in the blood [77].

Analysis of human upstream and downstream carotid plaque specimens demonstrated that plaques developed in downstream regions accumulated more neutrophils and exhibited enhanced expression of *NE* and *citH3*, thus confirming the pathological features of a vulnerable plaque phenotype. Additionally, the colocalisation of citH3 with neutrophil markers showed a greater distance between them in downstream plaques, suggesting increased NET extrusion in this region. Furthermore, the pro-atherosclerotic autoantibody anti-apolipoprotein A-1 (anti-ApoA-1 IgG) index positively correlated with the expression of *citH3* in plaques developed in downstream regions. Finally, the NET extrusion parameter (distance of citH3 from neutrophils) was increased in plaques derived from serum-positive anti-ApoA-1 patients compared with serum-negative patients [78].

**Figure 2:**
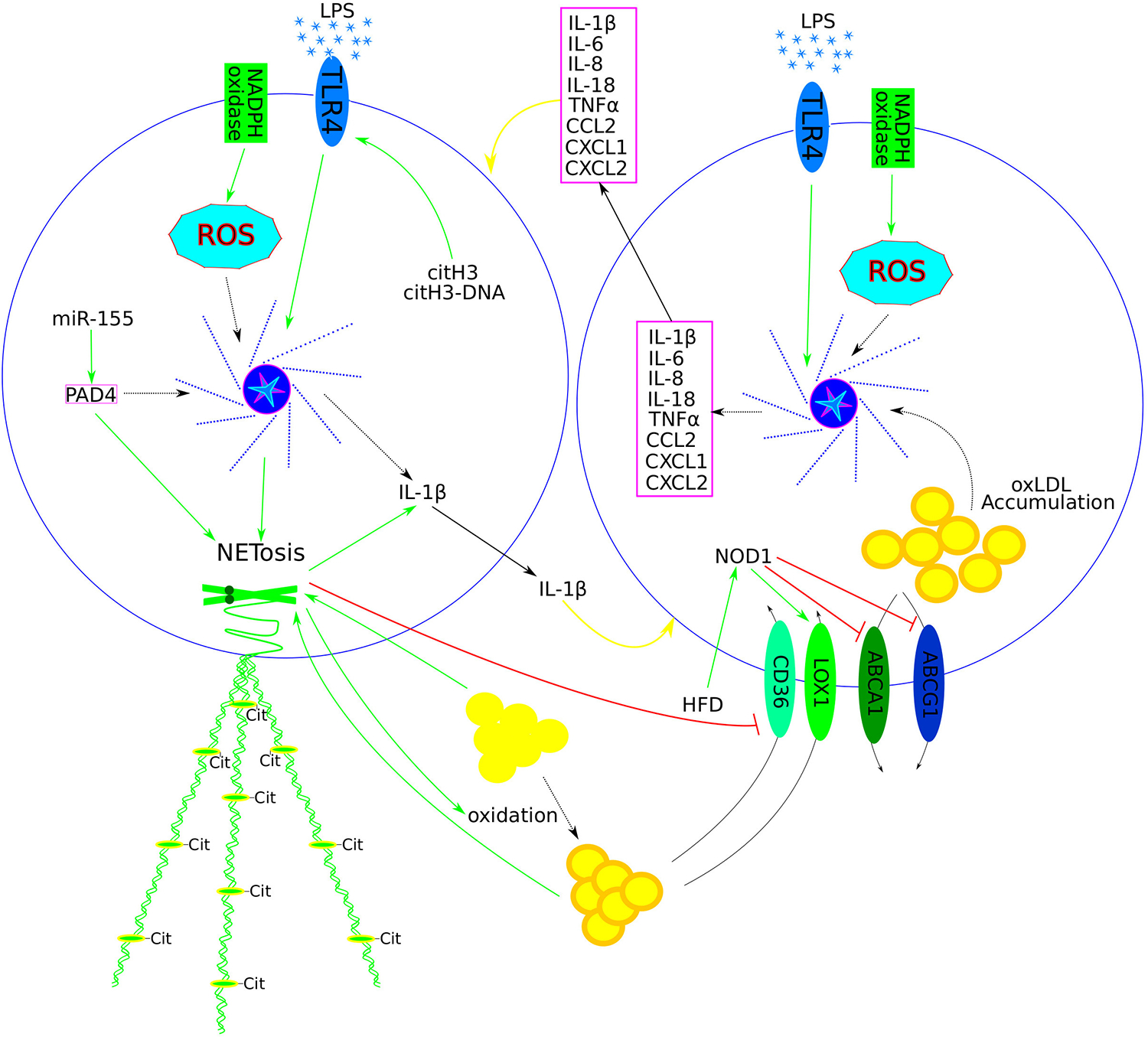
The central role of NLRP3 inflammasome activation and neutrophil-macrophage crosstalk in perpetuating a vicious cycle of inflammation and atherosclerosis progression. Components of NET (dead mitochondria, cell-free DNA, and others) prime macrophages and activate the NLRP3 inflammasome, facilitating the production and release of various pro-inflammatory cytokines (ILs, TNF*α*, and others (listed in the magenta box)), thereby activating neutrophils to form NET. Similarly, the NLRP3 inflammasome in neutrophils has been shown to produce and release IL-1*β* into the extracellular space, thus completing the vicious inflammatory circle (yellow arrows). Other signals (such as LPS and ROS) have also been shown to activate the NLRP3 inflammasome in both neutrophils and macrophages. In neutrophils, citH3 and the citH3-DNA complex up-regulate IL-1*β* expression and promote NET formation by acting on TLR4. MiR-155 has been identified as a positive regulator of PAD4, which subsequently positively regulates NET formation both directly and indirectly via the NLRP3 inflammasome. Both LDL and oxLDL promote NETosis, while NETosis facilitates the oxidation of LDL to oxLDL and down-regulates the expression of the oxLDL receptor CD36. HFD activates NOD1, which up-regulates the oxLDL receptor LOX1 and down-regulates cholesterol efflux genes (ABCA1/G1), thereby increasing oxLDL uptake and accumulation in macrophages. Intracellular oxLDL further increases pro-inflammatory cytokine levels via NLRP3 inflammasome stimulation and facilitates foam cell formation. Positive regulation is depicted with green arrows, negative regulation with red blunt lines, and dotted arrows represent missing steps in a multi-component signalling pathway. Abbreviations: NOD-, LRR- and pyrin domain-containing protein 3 (NLRP3); tumour necrosis factor alpha (TNF*α*); interleukin (IL); lipopolysaccharide (LPS); reactive oxygen species (ROS); citrullinated H3 (citH3); toll-like receptor 4 (TLR4); peptidyl arginine deiminase 4 (PAD4); oxidised low-density lipoprotein (oxLDL); a cluster of differentiation 36 (CD36, or fatty acid translocase (FAT)); nucleotide-binding oligomerisation domain 1 (NOD1); lectin-type oxidised LDL receptor 1 (LOX1); ATP-binding cassette transporter A1 (ABCA1); ATP-binding cassette transporter G1 (ABCG1)

Recent research has demonstrated that the pro-inflammatory role of NET depends on chromatin fragmentation, citrullination, and the close interaction between citrullinated histones and DNA. Evaluations of NET formation in human neutrophil cultures treated with a PAD4 inhibitor and in *ApoE*/*PAD4* deficient mice fed an HFD showed that citrullination is not essential for NET formation. Nucleosomes, citH3, and citH3 complexed with NET DNA up-regulated *IL-1β* expression. Further experiments with TLR blocking antibodies revealed that histones bind to and activate TLR4, with citrullination potentiating this interaction (Fig. 2). NET DNA stimulated the translocation of TLR4 to the cell surface, facilitating the recognition of extracellular chromatin. Treatment of *ApoE*-deficient mice with chromatin-blocking antibodies did not affect blood cholesterol and triglyceride levels but reduced the size of atherosclerotic lesions. A similar effect on plaque size was observed in *ApoE/PAD4*-deficient mice. These results demonstrated that extracellular chromatin is a major driver of local sterile inflammation that promotes atherosclerosis. Chromatin components play different roles: histones bind to and activate TLR4 to induce IL-1*β* transcription in mononuclear cells, while chromatin DNA regulates intracellular TLR4 localisation to promote chromatin recognition [79].

Experiments on hypercholesterolaemic mice showed that LPS stimulation promoted myeloid cell accumulation, thus increasing atherosclerotic lesion size. Interestingly, LPS treatment caused NET deposition along the arterial lumen, while inhibition of NET release prevented LPS-stimulated lesion growth. Furthermore, the application of histone H2a-specific antibodies or *in silico*-designed cyclical peptides identified NET-resident histone H2a as an agent responsible for monocyte adhesion in a receptor-independent, surface charge-dependent manner [80].

In another research on hypercholesterolaemic mice with established lesions, the blockade of histone H4 with antibody reduced plaques vulnerability and increased lesional SMC content. Furthermore, administration of the histone inhibitory peptide (HIPe), which disturb the interaction of histone H4 with SMC plasma membrane and prevents induction of SMC death, was proposed as a potent therapeutic strategy to prevent histone H4-driven cytotoxicity. Thus, the continuous HIPe administration to atherosclerotic mice increased lesional SMC content and increased lesion stability, thereby confirming its therapeutic value [81].

Histones play a pivotal role in NET formation and their involvement in atherosclerosis is significant. During NETosis, histones are citrullinated and released into the extracellular matrix, where they contribute to EC damage, inflammation, and tissue injury. Thus, histone H4 was associated with SMC cytotoxicity and increased plaque vulnerability. Citrullinated histones, particularly citH3, facilitate NET extrusion and enhance pro-inflammatory responses, promoting atherogenesis. Furthermore, histones interact with TLR4, driving local inflammation and exacerbating plaque progression. Thus, targeting histone-mediated mechanisms offers potential therapeutic avenues for mitigating NET-associated atherosclerosis.

## 3 PAD4 and Its Contribution to Neutrophil Extracellular Traps: Implications for Cardiovascular Inflammation

NET formation requires PAD4 to convert arginyl residues in histones of chromatin to citrulline [54]. PAD4 inhibition has been shown to prevent NETosis, reduce atheroma burden, and decrease neutrophil and monocyte recruitment to arteries [120]. However, since these results were obtained using chloramidine, an unspecific inhibitor affecting all PADs (PAD1 to 4), the precise role of PAD4 in NET formation and atherothrombosis remains unclear. Further studies involving the transplantation of *Pad4*^−*/*−^ bone marrow into male HFD-fed (5 and 10 weeks) *Ldlr*^−*/*−^ mice indicated no effect on fatty streak formation, plaque size, or inflammation parameters (Table 1). Nevertheless, *Pad4*^−*/*−^ bone marrow deficiency combined with DNase I administration disrupted NET, thereby preventing endothelial injury and thrombus formation within atheromata, mimicking features of superficial erosion. In total, the results demonstrated that NET formation depends on PAD4, while the formation and progression of atheromatous plaques in hypercholesterolaemic mice are PAD4 independent [82].

Conversely, transplantation of *Pad4*^−*/*−^ bone marrow into *ApoE*^−*/*−^ male mice did not affect atherosclerotic plaques after 4 weeks of HFD but resulted in reduced plaque size, decreased necrotic core area and collagen deposition after 10 weeks of HFD compared to control *ApoE*^−*/*−^ mice. This deficiency was associated with upregulated aortic expression of pro-inflammatory genes *CCL2* and *iNOS* and a higher content of M1 (pro-inflammatory) phenotype macrophages, which exhibited increased expression of *IL-6* and *iNOS*. M1 macrophages internalised less oxLDL *in vitro*, whereas M2 macrophages showed enhanced oxLDL uptake, correlating with increased expression of the oxLDL receptor *CD36* (Fig. 2). These findings suggest that *PAD4* deficiency affects the pro-inflammatory macrophage phenotype and impedes atherosclerotic plaque progression [83].

Contradictory results were observed in female *ApoE*^−*/*−^ mice fed HFD for 6 weeks, where myeloid-specific deletion of PAD4 reduced NET formation, atherosclerotic plaque area, and levels of pro-inflammatory mediators (IL-1*β*, IL-17A, CCL2, CXCL1, and CXCL2) in the aorta. *In vitro* experiments confirmed that NET presence induced IL-1*β*, CCL2, CXCL1, and CXCL2 production by macrophages. Treatment with DNase I decreased NET formation and ameliorated atherosclerosis in *ApoE*^−*/*−^ *PAD4* KO mice, reducing levels of IL-1*β*, TNF*α*, CCL2, CXCL1, and CXCL2 under both *in vitro* and *in vivo* conditions [84]. Overall, these results suggest potential sex-specific mechanisms in PAD4 involvement in NET formation and atherosclerosis.

Interesting results were obtained in a mouse model of acute MI, where the impact of NET and *PAD4* deficiency on macrophage polarisation and inflammation was investigated. Under M1-polarising conditions in macrophage *in vitro* cultures, NET-enriched supernatants from *in vitro* activated bone marrow-derived neutrophils suppressed the expression of inducible nitric oxide synthase (*iNOS*), *IL-6*, and *TNFα*, while upregulating the expression of *arginase I* (*Arg I*), a marker of the M2 phenotype, and increasing the secretion of anti-inflammatory IL-10. This suggests that NET promotes macrophage polarisation towards a reparative M2 phenotype. These effects were not observed following DNase I treatment or in *Pad4*^−*/*−^ cell cultures. In fibroblast cultures, under normoxic conditions, NET suppressed *collagen-1/3* expression, and under hypoxic conditions, only *collagen-3* was suppressed while transforming growth factor*β* (*TGFß)* expression was increased. *Pad4*^−*/*−^ mice with induced acute myocardial infarction (AMI) exhibited the absence of NET after *ex vivo* stimulation, increased levels of circulating cell-free DNA (cfDNA), mitochondrial DNA, and cardiac troponin. These mice also showed reduced cardiac expression of *IL-6*, *IL-10*, and M2 marker genes, along with increased *TNFα* expression, indicating overwhelming inflammation. At day 1 post-AMI, *Pad4*^−*/*−^ mice had increased end-diastolic volume and thinning of the left ventricular wall. However, by day 21, they demonstrated improved cardiac function with increased ejection fraction, stroke volume, and reduced left ventricular end-diastolic diameter, suggesting reduced cardiac hypertrophy and remodelling. These results demonstrate that NET is required for macrophage polarisation towards an M2 anti-inflammatory phenotype, and *PAD4* deficiency exacerbates acute inflammation and tissue damage following AMI induction. Nevertheless, at later stages after AMI, *Pad4*^−*/*−^ mice showed enhanced cardiac regeneration. This implies that *PAD4* deficiency triggers compensatory mechanisms, highlighting the need for further studies to elucidate the role of NET in inflammation and cardiac healing in post-AMI models, particularly with alternative strategies to prevent NET formation, such as DNase I infusion [85].

*In vitro* experiments have shown that CXCL1 treatment induces the translocation of PAD4 from the nucleus to the cytoplasm, thereby stimulating the adhesion of Human promyelocytic leukemia cells (HL-60 cells) and neutrophils to HUVEC. These effects, however, were reversed by the application of PAD4 inhibitor Thr-Asp-F-amidine trifluoroacetate salt (TDFA), a PAD4 inhibitor, or *PAD4* knockdown via siRNA. Additionally, *PAD4* knockdown or inhibition decreased the expression of *β2-integrin* and reduced F-actin polymerisation activated by CXCL1. Protein disulphide isomerase A1 (PDIA1) was identified as being citrullinated following CXCL1 treatment, suggesting that PAD4 translocation to the cytoplasm contributes to this modification. Notably, *PDIA1* knockdown or inhibition reduced the adhesion of HL-60 cells to HUVEC, as well as *β2-integrin* expression and F-actin polymerisation. Future studies are needed to further elucidate the role of citrullinated PDIA1 in neutrophil adhesion and atherosclerosis development [86].

Recent research has highlighted the positive role of miR-155 in regulating PAD4-dependent NET generation. Transfection with antagomiR-155 decreased PMA-induced *PAD4* expression and NET formation. Further investigation revealed that miR-155 directly binds to PAD4 mRNA via AU-rich elements in the 3′-UTR region. These findings suggest that targeting miR-155 may be a potential therapeutic strategy to inhibit excessive NET generation in inflammatory diseases [87].

The involvement of the canonical NLRP3 inflammasome in NET formation, and conversely, PAD4-dependent NLRP3 inflammasome formation in neutrophils, has recently been demonstrated. Under sterile conditions (i.e., without LPS stimulation), neutrophil activation induces NLRP3-dependent assembly of apoptosis-associated speck-like protein containing a CARD (ASC) and subsequent caspase-1 cleavage. Experiments with global and haematopoietic cell-specific *PAD4* knockout models revealed that PAD4 supports ASC speck formation by regulating NLRP3 and ASC protein levels. As anticipated, genetic ablation or pharmacological inhibition of NLRP3 reduced NET formation in both mouse and human neutrophil cell cultures and diminished NET density in thrombi in a stenosis-induced mouse model of deep vein thrombosis [88].

In summary, PAD4 plays a pivotal role in NET formation and atherosclerosis development. PAD4-mediated citrullination of histones is crucial for NET formation, and its inhibition reduces NETosis, atheroma burden, and inflammatory cell recruitment to arteries (Table 1). While some studies report *PAD4* deficiency leading to reduced atherosclerotic plaque progression and altered macrophage phenotype, others highlight the complexity of PAD4’s role, suggesting sex-specific differences and potential compensatory mechanisms. Additionally, PAD4’s interactions with CXCL1, PDIA1, and miR-155, as well as its involvement in NLRP3 inflammasome activation, underscore its multifaceted impact on inflammation and atherosclerosis. Future research should further explore these mechanisms and investigate alternative methods to modulate NET formation beyond PAD4 inhibition, aiming to refine therapeutic strategies for atherosclerosis and related inflammatory diseases.

## 4 Cholesterol’s Impact on Neutrophil Extracellular Traps: Implications for Atherosclerosis Progression

Cholesterol efflux pathways are known to suppress the inflammatory response in myeloid cells, whereas cholesterol accumulation exacerbates inflammation, thereby promoting atherosclerosis [121]. The ATP-binding cassette transporters A1 and G1 (ABCA1/G1) are the major transporters mediating cholesterol efflux to apolipoprotein A-1 and HDL [122]. Low levels of ABCA1/G1 in monocytes/macrophages and reduced plasma HDL levels are associated with an increased risk of cardiovascular diseases [123,124]. Accordingly, patients with Tangier Disease, who carry loss-of-function mutations in *ABCA1*, exhibit an elevated risk of cardiovascular disease mortality [125]. Recent research has demonstrated a close interaction between neutrophils and macrophages through NLRP3 inflammasome activation and NETosis in atherosclerotic plaques.

Thus, the transplantation of *Abca1/g1*-deficient bone marrow into *Ldlr*^−*/*−^ mice resulted in increased plasma IL-18 levels and induced IL-1*β* and IL-18 secretion in splenocytes (Table 1). These effects were reversed by *Nlrp3* or *Caspase-1/11* deficiency, suggesting the involvement of the NLRP3 inflammasome. Accordingly, *Nlrp3* or *Caspase-1/11* deficiency decreased atherosclerotic lesion size in bone marrow *Abca1/g1*-deficient *Ldlr*^−*/*−^ mice. Furthermore, bone marrow *Abca1/g1* deficiency enhanced Caspase-1 cleavage in the spleen, and increased neutrophil accumulation, NET formation, and cholesterol accumulation in macrophages and monocytes within atherosclerotic plaques. These effects were similarly reversed by *Nlrp3* or *Caspase-1/11* deficiency, indicating that inflammasome activation promotes neutrophil recruitment and NETosis in atherosclerotic plaques. Blood samples from Tangier Disease patients showed increased myeloid cholesterol content and elevated plasma IL-1*β* and IL-18 levels [89]. Further research specifies that macrophage-specific *Abca1/g1* deficiency is responsible for inflammasome activation and NETosis induction in plaques. The application of an IL-1*β*-neutralising antibody or NLRP3 inhibitor suppressed NETosis, suggesting that these effects are dependent on the presence of IL-1*β* and NLRP3 [90].

Splenic nucleotide-binding oligomerisation domain 1 (NOD1) regulates neutrophil accumulation and activity in mice fed an HFD, thereby influencing atherogenic progression. In *ApoE*^−*/*−^ mice, HFD activated splenic NOD1, which was associated with increased NET release. However, NET formation was reduced in *ApoE*^−*/*−^*Nod1*^−*/*−^ mice, accompanied by decreased neutrophil recruitment in the spleen and a reduced atherosclerotic lesion area. Notably, splenic artery ligation reduced the atherogenic burden in *ApoE*^−*/*−^ mice, an effect that was lost in *ApoE*^−*/*−^*Nod1*^−*/*−^ mice. Additionally, the absence of NOD1 was associated with decreased levels of CXCL12 in the lesion area, although splenic artery ligation reduced CXCL12 levels independently of NOD1. NOD1 also regulated splenic lipid metabolism. HFD-fed *ApoE*^−*/*−^*Nod1*^−*/*−^ mice exhibited higher levels of LDL and increased expression of cholesterol efflux genes (*ABCA1/G1*), alongside reduced expression of the oxidised LDL receptor *LOX1*. Consequently, spleen lipid content decreased in *ApoE*^−*/*−^*Nod1*^−*/*−^ mice and spleen ligation enhanced HDL and non-esterified fatty acid levels. These findings indicate that HFD-induced NOD1 activation partly regulates neutrophil accumulation and activity in the spleen, thereby contributing to atherogenic progression. Moreover, HFD-mediated activation of splenic NOD1 is involved in lipid regulation and accumulation in the spleen [91]. Future research should also consider other humoral and cellular factors associated with spleen ligation.

Furthermore, hypercholesterolaemia has been shown to promote atherosclerotic plaque progression by impairing NET clearance, which is mediated by endonucleases such as DNase I and DNase IL3 (Fig. 1). The increase in systemic NET levels led to a rapid rise in serum DNase activity; however, in hypercholesterolaemic mice, this NET-induced DNase response was delayed, resulting in defective inflammation resolution. DNase I infusion in *ApoE*^−*/*−^ mice fed an HFD with advanced atherosclerosis reduced plaque NET content, plaque necrosis area, and increased collagen content. Moreover, the application of tauroursodeoxycholic acid to alleviate endoplasmic reticulum stress rescued the hypercholesterolaemia-induced delay in the NET-induced DNase response, indicating that endoplasmic reticulum stress plays a role in regulating DNase response. These results were corroborated in patients with hypercholesterolaemia, who showed increased systemic extracellular DNA levels and decreased plasma DNase activity, suggesting a potential therapeutic benefit in targeting NET-induced DNase response to promote inflammation resolution and stabilise atherosclerotic plaques [92].

Cholesterol efflux pathways play a crucial role in modulating inflammatory responses and atherogenesis. Dysregulation of cholesterol transporters, such as ABCA1/G1, leads to increased cholesterol accumulation in macrophages and enhanced inflammatory responses, which in turn promote NET formation and progression of atherosclerosis. Deficiencies in these transporters and associated inflammasome activation exacerbate neutrophil recruitment and NETosis within atherosclerotic plaques. Moreover, impaired NET clearance due to hypercholesterolaemia further contributes to persistent inflammation and plaque instability. These findings underscore the importance of cholesterol metabolism in regulating NET formation and its impact on atherosclerosis, highlighting potential therapeutic targets for managing cardiovascular diseases.

## 5 The Influence of LDL and HDL on Neutrophil Extracellular Trap Formation in Atherosclerosis

The role of different lipoproteins and their modified products has been widely and extensively studied in relation to CVD development. Despite the inconclusive role of oxLDL in atherosclerosis initiation, elevated levels of oxLDL are considered a risk factor for the development and progression of CVD. Accordingly, oxLDL is commonly used in experimental models to induce atherosclerosis by activating various pro-atherogenic factors [8].

Recent research has demonstrated that oxLDL affects NET formation, thereby promoting vascular inflammation and atherosclerosis progression (Table 1). Specifically, the presence of oxLDL, but not native LDL, enhanced NET formation in PMA-induced cultures of HL-60-derived neutrophils. Furthermore, transferring the culture media containing NET formed by HL-60-derived neutrophils to human aortic endothelial cells (HAEC) increased the expression of *matrix metalloproteinase-1* (*MMP-1*) and induced morphological changes in HAEC [93]. Similar NET-promoting effects were observed with oxidised phosphatidylcholines, lysophosphatidylcholine—a pro-inflammatory lipid produced during LDL oxidation—and the electronegative subfraction of LDL (LDL–), whereas HDL acted in the opposite manner by suppressing NET formation [94].

Conversely, NET increased the expression of *MMP-9* and pro-inflammatory cytokines *IL-1β*, *IL-6*, and *TNFα*, while decreasing the expression of the long-chain free fatty acid receptor *CD36*. They also enhanced the incorporation of oxLDL into human and murine M1 macrophages, suggesting that NET may promote foam cell formation and plaque vulnerability [95].

Recent studies have investigated the role of NET in atherosclerosis and thrombosis using mice with haematopoietic *Lymphocyte Adaptor Protein* (*LNK)* deficiency. *LNK* (also known as *SH2B Adaptor Protein 3*) is primarily expressed in haematopoietic and EC, where it functions as a negative regulator of cytokine signalling and cell proliferation [96]. Deletion of *LNK* in mice causes expansion of haematopoietic stem cells, increased myelopoiesis, thrombocytosis, and leukocytosis. In humans, the common T risk single nucleotide polymorphism rs3184504 (T allele, R262W) is associated with increased platelet and neutrophil counts, CAD, and thrombotic stroke [126]. Thus, bone marrow from wild-type, *Lnk*^−*/*−^, *Pad4*^−*/*−^, and *Lnk*^−*/*−^ *Pad4*^−*/*−^ mice was transplanted into HFD-fed *Ldlr*^−*/*−^ mice. As expected, recipients of *Lnk*^−*/*−^ bone marrow exhibited prominent NETosis, accompanied by accelerated carotid artery thrombosis, increased lesion size, and IL-1*β* production; these effects were reversed by *PAD4* deficiency. Oxidised phospholipids released from *Lnk*^−*/*−^ platelets were identified as factors responsible for accelerated NETosis. Increased NETosis was also observed in experiments with human induced pluripotent stem cell–derived LNK(TT) neutrophils, thus confirming the clinical relevance of the results [97].

In summary, oxLDL plays a significant role in promoting atherosclerosis through its effects on NET formation. OxLDL enhances NET production, leading to increased vascular inflammation and atherosclerotic progression, whereas HDL suppresses NET formation. NET contribute to foam cell formation and plaque vulnerability by promoting the incorporation of oxLDL into macrophages and increasing pro-inflammatory cytokine levels. Conversely, the role of NET in atherosclerosis is further emphasised by findings in mice with haematopoietic *LNK* deficiency, which demonstrate accelerated NETosis and enhanced atherogenic processes. Overall, targeting oxLDL and NET-related pathways may offer potential therapeutic strategies for managing atherosclerosis and cardiovascular diseases.

## 6 The Impact of Radiation on NET Formation and Its Contribution to Atherosclerosis Progression

Exposure to radiation can stimulate the development of atherosclerosis and cause damage to various organs, including the liver, heart, lungs, haematopoietic system, and gastrointestinal tract [127,128]. Radiation-mediated vascular damage increases immune cell infiltration into tissues, production of pro-inflammatory cytokines and ROS, and dysregulation of lipid metabolic pathways [129]. Recent studies have reported a correlation between radiation dosage and the number of adverse coronary events, suggesting its role in the development of CVD [130]. Exposure of neutrophils derived from healthy donors to very low doses of *γ*-radiation (0.5–1 Gy) induces NET formation in an oxidative stress, nicotinamide adenine dinucleotide phosphate oxidase (NADPH oxidase) activity, and IL-8-dependent manner (Table 1). Consequently, an increase in circulating NET has been observed in patients undergoing radiotherapy [98].

In atherosclerotic *Ldlr*^−*/*−^ mice, exposure to low-dose (0.5 or 1 Gy) chronic radiation (over 16 weeks) increased levels of total plasma cholesterol, LDL-C, and triglycerides (TG), as well as lipid-laden plaque size, ROS levels, IL-1*β*, and TNF*α* in the aortas and spleens. Additionally, radiation in a dose-dependent manner increased the infiltration of neutrophils and monocytes into the spleens of *Ldlr*^−*/*−^ mice, while reducing lymphocyte levels. These results confirm that chronic radiation exposure exacerbates atherosclerosis in *Ldlr*^−*/*−^ mice through increased production of pro-inflammatory mediators [99]. Similarly, long-term exposure to low-dose radiation (accumulated dose of 0.2 Gy) increased vascular stiffness and aortic lesions in HFD-fed *ApoE*^−*/*−^ mice. Specifically, radiation, rather than HFD, aggravated HFD-induced atherosclerosis by activating the cyclic GMP-AMP synthase-stimulator of interferon genes (cGAS-STING) signalling pathway through phosphorylation of TANK-binding kinase 1 (TBK1) and interferon regulatory factor 3 (IRF-3), leading to increased expression of pro-inflammatory mediators *IL-1β*, *IL-18*, *IFNα*, and *IFNβ*, thereby promoting atherosclerotic plaque formation [100]. These findings align with previous studies highlighting the active involvement of the cGAS-STING signalling pathway in atherosclerosis progression [101,102].

However, experiments on HFD-fed mice irradiated with 0.5 Gy showed contrasting results. Radiation increased the number of neutrophils but reduced NET concentration compared to the control group, while NET concentration was elevated in the HFD-fed group. Moreover, plaque size increased in the HFD-fed group but significantly decreased in the irradiated group compared to the control. These results confirm the central role of neutrophils and NET in atherosclerosis but suggest that radiation may act as a rather atheroprotective factor. The discrepancy in results may be attributed to differences in radiation dose, treatment duration (5 days), or scheme (5 days of treatment/2 rest days), the overall physical condition of the mice, or interactions between radiation exposure and other atherogenic risk factors [103].

Exposure to radiation has been implicated in accelerating atherosclerosis and exacerbating vascular damage across various organ systems (Table 1). Chronic low-dose radiation increases plasma cholesterol, lipid-laden plaque size, and pro-inflammatory cytokine levels, which contributes to aggravated atherosclerosis in animal models. Specifically, radiation activates the cGAS-STING signalling pathway, further promoting inflammatory responses and plaque formation. However, some studies suggest a paradoxical atheroprotective effect of radiation, potentially due to variations in radiation dose, exposure duration, and interaction with other atherogenic factors. Further research is needed to reconcile these findings and elucidate the mechanisms underlying the differential impact of radiation on atherosclerosis.

## 7 MiR-146a and Its Implications in Neutrophil Extracellular Trap Formation and Atherosclerosis

MicroRNAs (miRNAs) are small, non-coding RNAs that post-transcriptionally regulate gene expression, thereby participating in various signalling pathways, including development, immune response, and the progression of various diseases [131]. MiR-146a is well-characterised as a negative regulator of Nuclear Factor Kappa B (NF-*κ*B) activity through its ability to target upstream adaptor proteins, such as TNF receptor-associated factor 6 (TRAF6) and interleukin receptor-associated kinase 1 (IRAK1) [132]. Increased expression of *miR-146a* has been detected in human atherosclerotic plaques, and polymorphisms in the miR-146a precursor have been associated with an increased risk of coronary artery disease [133]. The role of miR-146a in atherosclerosis progression has been extensively studied, with often contradictory results, showing miR-146a as either a positive, a negative, or having no significant role in atherogenesis [134–137].

Recent research has demonstrated the involvement of miR-146a in regulating NET formation and thrombosis, but not in atherosclerotic plaque formation. Experiments on *Ldlr*^*−/−*^ mice fed a HFD with transplanted bone marrow from *miR-146a*^*−/−*^ mice, and stimulated with lipopolysaccharide, showed that *miR-146a*^*−/−*^ deficiency increased NETosis in both models (Table 1). Furthermore, *miR-146a*^*−/−*^ mice exhibited reduced carotid occlusion time and elevated levels of NET in thrombi following FeCl_3_-induced thrombosis. DNAse I infusion abolished arterial thrombosis in both wild-type and *miR-146a*^*−/−*^ mice. Interestingly, *miR-146a*^*−/−*^ mice displayed an aged neutrophil phenotype, which primed these cells for activation, making them more prone to NET formation regardless of the stimulus, and thus contributing to thrombosis [104]. As a possible mechanism, miR-146a has been proposed to induce NET formation by targeting and down-regulating superoxide dismutase 2 (SOD2), thereby promoting ROS production via the miR-146a/SOD2/ROS pathway (Fig. 1) [105].

In summary, miR-146a has a complex and multifaceted role in cardiovascular pathology, with its effects on atherosclerosis remaining contentious. While some studies suggest its involvement in atherosclerosis, recent research highlights its more defined role in regulating NET formation and thrombosis. *miR-146a* deficiency is associated with increased NETosis and heightened thrombotic risk in mouse models, attributed to an aged neutrophil phenotype and elevated ROS production through the miR-146a/SOD2/ROS pathway. These findings suggest that miR-146a’s primary impact may be on thrombotic processes rather than plaque formation, positioning it as a potential therapeutic target for managing NET-driven thrombosis. Further investigations are needed to clarify its precise role in atherosclerosis and to explore its therapeutic potential.

## 8 NET Components as Biomarkers in Atherosclerosis and Related Diseases

Given the involvement of NET in the pathological processes of atherosclerosis, their components have emerged as potential biomarkers for assessing disease severity and predicting clinical outcomes in atherosclerosis and associated complications [138,139]. Various NET components, such as double-stranded DNA, MPO–DNA complexes, modified histones, and NE, are commonly measured to evaluate NET activity. Additionally, IL-8 and IL-33 have been suggested as primary pro-inflammatory mediators promoting NET in atherosclerosis progression [140,141].

Advanced atherosclerotic plaque causes narrowing of arteries (stenosis), reducing blood supply and potentially leading to stroke (in cases of carotid stenosis) or heart attack (in cases of coronary artery disease). NET in plaques are associated with an increased risk of plaque rupture and subsequent thrombosis, particularly by enhancing the accumulation of pro-thrombotic molecules, including fibrinogen and von Willebrand factor [106]. Thus, patients with symptomatic carotid stenosis exhibited higher levels of NET markers compared to asymptomatic controls and healthy subjects (Table 1) [107]. Another study involving 182 patients from the Plaque At RISK (PARISK) cohort found a positive association between the vulnerability index and NET levels (assessed via myeloperoxidase-DNA complexes), though no such association was observed in the overall population. Notably, this positive association was driven by intra-plaque haemorrhage, lipid-rich necrotic core, and ulceration [108]. Additionally, the level of PAD4 in local arterial blood was associated with the neutrophil to lymphocyte ratio, HDL, TG/HDL ratio, and ulceration in 39 patients with carotid artery stenosis, suggesting PAD4 as a potential biomarker of plaque instability [109].

Clinical characterisation of 243 patients with acute ischaemic stroke revealed higher plasma levels of NET compared to healthy subjects. NET levels were elevated in patients over 65 years of age and those with a history of atrial fibrillation (AF), cardioembolic stroke, high glucose levels, and severe stroke scores. Among the NET markers used (cell-free DNA, nucleosomes, and citH3), citH3 was identified as the most specific marker, independently associated with AF and all-cause mortality at one-year follow-up [110]. Moreover, in stroke patients, NET were shown to convert EC to a procoagulant phenotype, thereby increasing endothelial barrier dysfunction and thrombogenicity [111]. Furthermore, double-stranded DNA levels were related to adverse clinical outcomes after two years but were only weakly associated with hypercoagulability in patients with stable coronary artery disease [112]. However, circulating NET levels were not associated with type 1 diabetes mellitus (T1DM) or the presence of coronary artery disease in patients with T1DM [142].

Finally, a significant association has been established between NET and acute STEMI, which is a leading cause of death in patients with CAD [113]. Elevated levels of double-stranded DNA (dsDNA) from the culprit site were linked to a higher rate of long-term major adverse cardiovascular events (MACE) in STEMI patients [114–116]. Additionally, dsDNA levels were correlated with plasma glucose levels in both acute and stable conditions, suggesting glucose-stimulated NETosis in STEMI patients [143]. CitH3, another NET marker, was associated with increased levels of monocyte chemoattractant protein 1 (MCP-1) at the culprit site in STEMI patients. Furthermore, an increasing MCP-1 gradient correlated with fibrocyte accumulation at the culprit site, indicating its role in both inflammation and scar formation [117]. Similar findings were reported in another study, where NET markers (dsDNA and citH3) in STEMI patients positively correlated with infarct size and left ventricular dysfunction at follow-up, confirming the role of NET as mediators of fibrotic remodelling by stimulating fibrocytes [144].

Analysis of NET (MPO and citH3) in thrombi from STEMI patients demonstrated that the presence of NET was associated with the occurrence of MACE within the first 30 days after MI, primarily due to cardiac deaths and stent thrombosis [118]. In another study, high levels of NET in thrombi from STEMI patients were associated with systemic inflammation (indicated by high levels of C-reactive protein) and less advanced atherosclerosis (characterised by smaller lipid arcs and larger flow areas) [119].

NET and their components have emerged as promising biomarkers for CVD, particularly in assessing the severity and prognosis of atherosclerosis and its complications. Elevated levels of NET markers, such as double-stranded DNA, citH3, and MPO, are associated with increased risk of MACE and can provide insights into disease progression and response to treatment (Table 1). Specifically, NET play a significant role in acute conditions such as STEMI, where their presence correlates with adverse outcomes and systemic inflammation. Despite the promising potential, further research is needed to fully elucidate their clinical utility and to develop targeted therapies for NET-related pathological processes in CVD.

## 9 Limitations and Future Directions

The existing research on NET and their role in CVD, including atherosclerosis and MI, has several limitations that must be addressed to enhance the validity and applicability of findings. One significant limitation is the variability in experimental design and model systems used across studies. For example, different studies employ various animal models (such as *Ldlr*^*−/−*^ or *ApoE*^*−/−*^ mice) and experimental conditions, which can lead to discrepancies in results. Additionally, the use of diverse chemicals and inhibitors, such as chloramidine and TDFA, can introduce variability, as these compounds may not exclusively target PAD4 or other specific pathways, complicating the interpretation of their effects on NET formation and cardiovascular outcomes.

Another issue is the contradiction in findings regarding the role of PAD4 and miR-146a in NET formation and atherosclerosis. Some studies suggest a positive role of PAD4 in promoting NET formation and atherosclerosis, while others indicate its inhibitory effects or lack of involvement. Similarly, miR-146a has been reported to both enhance and inhibit NET formation in different contexts. These inconsistencies may stem from differences in experimental conditions, methodologies, or the specific models used, highlighting the need for standardisation in experimental protocols.

Future research should focus on addressing these limitations by employing more uniform experimental designs and using well-characterised models to ensure reproducibility and comparability of results. Additionally, studies should consider using more specific inhibitors or genetic models to precisely target and validate the role of specific pathways, such as PAD4, in NET formation and cardiovascular disease. Moreover, incorporating diverse patient populations and longitudinal studies could help clarify the clinical relevance of NET components as biomarkers and their impact on disease progression and outcomes.

To resolve contradictions and provide a clearer understanding of NET in cardiovascular diseases, future research should aim to integrate findings from different models and approaches. This includes validating results across various experimental conditions and exploring the underlying mechanisms driving the observed discrepancies. Furthermore, advancing our understanding of how NET interact with other components of the immune system and vascular biology will be crucial for developing targeted therapeutic strategies and improving patient management.

It is important to note that in addition to neutrophils, other cells—macrophages, eosinophils, leukocytes (B cells, T cells, monocytes, and natural killer cells)—were also shown to produce ETs participating in response to pathogens, various inflammatory and auto-immune disease, such as asthma, lung diseases, vasculitis, autoimmune kidney diseases, rheumatoid arthritis, psoriasis, and cancer [145–147]. In the context of atherosclerosis, recent analysis of thrombosed plaques derived from patients who died from myocardial infarction demonstrated presence of neutrophil, macrophage, mast cell and eosinophil extracellular traps. The NET were prominent in early thrombosis, while the macrophage traps were more abundant in late thrombosis. The numbers of mast cell- and eosinophil-derived traps were lower compared to NET, while the most eosinophil-derived traps were present in lytic thrombi and mast cell-derived traps—in organised thrombi [148]. Accordingly, advancing our knowledge about the role of different cellular sources of extracellular traps during development and progression of atherosclerosis is vital for invention of novel strategies in the treatment of CVD.

## 10 Conclusion

This manuscript offers an extensive examination of NET and their multifaceted role in atherosclerosis and cardiovascular diseases. NET is crucial to understanding the pathogenesis of these conditions, as they impact both inflammatory and thrombotic processes. Radiation exposure has been demonstrated to enhance NET formation, which exacerbates inflammation and contributes to atherosclerotic plaque instability. The mechanisms by which radiation promotes NETosis involve increased oxidative stress and subsequent activation of neutrophils. Histones play a pivotal role in the formation and stability of NET. Citrullinated histones, in particular, are integral to NET structure and function. Their presence in atherosclerotic plaques suggests that histone modifications are critical in modulating NET-induced inflammation and thrombosis. The interplay between lipoproteins and NET underscores a complex relationship between lipid metabolism and atherosclerosis. Elevated levels of LDL cholesterol and decreased HDL cholesterol are associated with increased NET formation and atherosclerotic plaque progression. This relationship highlights the need for targeted lipid management to mitigate NET-related vascular damage. PAD4, an enzyme responsible for citrullinating histones, is central to NET formation. Inhibition or genetic ablation of PAD4 affects NETosis, with implications for atherosclerosis and related conditions. The effects of *PAD4* deficiency on plaque stability and inflammation further illustrate its potential as a therapeutic target. NET components such as dsDNA, MPO-DNA complexes, and citrullinated histones have emerged as significant biomarkers for assessing disease severity and predicting clinical outcomes in cardiovascular diseases. Elevated levels of these biomarkers correlate with plaque instability, increased risk of thrombosis, and adverse cardiovascular events.

In summary, NET is integral to the development and progression of atherosclerosis and other cardiovascular diseases. They serve both as key drivers of inflammation and thrombosis and as valuable biomarkers for disease assessment. Understanding the various factors influencing NET formation, including radiation, histone modifications, lipid profiles, and PAD4 activity, is crucial for developing effective diagnostic and therapeutic strategies. Future research should focus on elucidating these relationships further and exploring novel interventions to manage NET-related vascular pathology.

## Figures and Tables

**Table 1:** The role of NET in atherosclerosis and associated cardiovascular diseases

Experimental model	Changes in NET components and atherosclerosis-relevant parameters	Potential therapeutic approaches	References
*In vivo*: HFD-fed *LDLR*^*−/−*^ mice, CXCL1 application and PAD4 inhibition	Increased neutrophil adhesion, NE, MPO, and citH3 levels in circulating neutrophils	CXCL1 and PAD4 inhibitors to reduce neutrophil adhesion and citH3 levels	[77]
*In vivo*: Human carotid plaque specimens	Increased neutrophil accumulation, NE, and citH3 expression in downstream plaques	Targeting NET extrusion and citH3 in vulnerable plaques	[78]
*In vitro*: Human neutrophil cultures; *In vivo*: *ApoE*/*PAD4*-deficient mice, HFD treatment	NET formation with chromatin fragmentation and citrullination, TLR4 activation by histones	Chromatin-blocking antibodies, PAD4 inhibition, TLR4 blockade to reduce inflammation and atherosclerotic plaque size	[79]
*In vivo*: Hypercholesterolaemic mice with LPS stimulation	NET deposition along arterial lumen, monocyte adhesion facilitated by histone H2a	Inhibition of NET release and histone H2a-targeted therapies	[80]
*In vivo*: Hypercholesterolaemic mice with established lesions	Reduced plaque vulnerability, increased SMC content	Histone H4 blockade, HIPe administration to stabilise plaques and prevent SMC death	[81]
*In vivo*: *Ldlr*^−*/*−^ mice with *PAD4*^−*/*−^ bone marrow, HFD-fed (5 and 10 weeks)	*PAD4* deficiency did not affect plaque size or inflammation but disrupted NET formation, preventing endothelial injury and thrombus formation	DNase I treatment to prevent NET formation	[82]
*In vivo*: *ApoE*^−*/*−^ male mice with *PAD4*^−*/*−^ bone marrow, HFD for 10 weeks	Reduced plaque size, necrotic core area, collagen deposition; increased expression of pro-inflammatory genes (*CCL2*, *iNOS*); M1 macrophages had increased *IL-6* and *iNOS* expression	Targeting pro-inflammatory M1 macrophages, *PAD4* deficiency	[83]
*In vivo*: *ApoE*^−*/*−^ mice, female, myeloid-specific *PAD4* deletion, HFD for 6 weeks	Reduced NET formation, atherosclerotic plaque area, and pro-inflammatory mediators (IL-1*β*, IL-17A, CCL2, CXCL1, CXCL2) in aorta	DNase I treatment, PAD4-specific therapies	[84]
*In vivo*: Acute MI in *Pad4*^−*/*−^ mice	*PAD4* deficiency reduced NET formation, exacerbated acute inflammation, and impaired macrophage polarisation towards M2 phenotype; increased cardiac damage in early stages, but improved cardiac regeneration later	PAD4 inhibition, DNase I infusion, modulation of macrophage polarisation	[85]
*In vitro*: CXCL1 treatment in HL-60 cells, *PAD4* knockdown via siRNA or PAD4 inhibitor TDFA	CXCL1-induced PAD4 translocation to cytoplasm, enhanced neutrophil adhesion, increased *β2-integrin* expression, F-actin polymerisation	PAD4 inhibition, *PAD4* knockdown, targeting citrullinated PDIA1 to reduce adhesion	[86]
*In vitro*: Transfection with antagomiR-155	Decreased *PAD4* expression, reduced NET formation	Targeting miR-155 as a therapeutic strategy to inhibit excessive NET generation	[87]
*In vivo* and *in vitro*: NLRP3 inflammasome activation in *PAD4*^−*/*−^ models	Reduced NLRP3-dependent ASC speck formation, diminished NET formation and NET density in thrombi	NLRP3 inhibition, PAD4-targeted therapies to regulate inflammasome activation	[88]
*In vivo*: *Ldlr*^−*/*−^ mice with *Abca1/g1*-deficient bone marrow	Increased plasma IL-18 levels, secretion of IL-1*β* and IL-18 from splenocytes, increased neutrophil accumulation, NET formation, and cholesterol accumulation in plaques	IL-1*β*-neutralising antibody, NLRP3 inhibitor	[89,90]
*In vivo*: *ApoE*^−*/*−^ mice fed HFD with *NOD1* deficiency	Reduced NET formation, decreased neutrophil recruitment, and atherosclerotic lesion area	Splenic artery ligation, targeting NOD1 and lipid metabolism	[91]
*In vivo*: Hypercholesterolaemic *ApoE*^−*/*−^ mice (HFD-fed)	Impaired NET clearance, delayed DNase response, increased systemic NET levels, increased plaque NET content, plaque necrosis area, and reduced collagen content	DNase I infusion, tauroursodeoxycholic acid to alleviate endoplasmic reticulum stress	[92]
*In vitro*: HL-60-derived neutrophils with oxLDL	Enhanced NET formation in PMA-induced cultures, transfer of NET to HAEC increased *MMP-1* expression, morphological changes in HAEC	HDL to suppress NET formation	[93,94]
*In vitro*: M1 macrophages with NET exposure	Increased *MMP9*, *IL-1β*, I*L-6*, *TNFα* expression; decreased *CD36* expression, enhanced incorporation of oxLDL into macrophages, foam cell formation	Targeting cytokine production or foam cell formation	[95]
*In vivo*: *Lnk*^−*/*−^ mice transplanted with various bone marrow	Increased NETosis, accelerated thrombosis, increased lesion size, IL-1*β* production, reversed by *PAD4* deficiency	PAD4 inhibitors, targeting oxidised phospholipids	[96,97]
*In vitro*: Neutrophils derived from healthy donors exposed to *γ*-radiation	NET formation induced in an oxidative stress, NADPH oxidase activity, and IL-8-dependent manner	Targeting oxidative stress pathways, NADPH oxidase inhibitors	[98]
*In vivo*: *Ldlr*^−*/*−^ mice exposed to chronic low-dose radiation (0.5–1 Gy)	Increased neutrophil infiltration and lipid-laden plaque size, increased ROS levels, IL-1*β* and TNF*α* production	Targeting IL-1*β*, TNF*α*, or ROS pathways to reduce atherosclerosis	[99]
*In vivo*: *ApoE*^−*/*−^ mice exposed to long-term low-dose radiation (0.2 Gy)	Increased vascular stiffness, aortic lesions, and pro-inflammatory mediators (*IL-1β*, *IL-18*, *IFNα*, *IFNβ*)	Inhibition of cGAS-STING pathway, targeting inflammation in plaques	[100–102]
*In vivo*: HFD-fed mice exposed to low-dose radiation (0.5 Gy)	Decreased NET concentration despite increased neutrophils, plaque size reduction compared to control	Assessing radiation as an atheroprotective factor, radiation dose management	[103]
*In vivo*: *Ldlr*^−*/*−^ mice fed a HFD with transplanted bone marrow from *miR-146a*^−*/*−^ mice, stimulated with LPS and FeCl_3_-induced thrombosis	Increased NETosis, reduced carotid occlusion time, elevated NET levels in thrombi, aged neutrophil phenotype	Targeting miR-146a or SOD2 to reduce NET formation and thrombosis	[104,105]
Patients with symptomatic carotid stenosis vs. asymptomatic controls	Elevated NET markers in symptomatic patients	Targeting NET markers to assess plaque instability and prevent thrombosis	[106,107]
PARISK cohort: 182 patients with varying plaque vulnerability	Positive association between NET levels and plaque vulnerability index (myeloperoxidase-DNA complexes)	Monitoring NET levels to assess plaque vulnerability and stability	[108]
39 patients with carotid artery stenosis	PAD4 levels associated with neutrophil to lymphocyte ratio, HDL, triglycerides (TG)/HDL ratio, and plaque ulceration	PAD4 as a potential biomarker for plaque instability	[109]
243 patients with acute ischemic stroke	Elevated NET levels in patients with acute stroke, particularly in those with AF, cardioembolic stroke, high glucose levels, and severe stroke scores	Using NET markers (citH3) to predict stroke outcomes and mortality risks	[110]
Patients with stroke	NET convert EC to a procoagulant phenotype, increasing thrombogenicity	Targeting NET to prevent endothelial dysfunction and thrombotic events	[111]
Stable CAD patients	Circulating NET levels weakly associated with hypercoagulability	NET-targeting therapies to reduce thrombotic risk in CAD	[112]
STEMI patients	Elevated dsDNA levels linked to MACE and long-term outcomes	Targeting dsDNA to reduce MACE in STEMI patients	[113–116]
STEMI patients	Increased citH3 levels associated with MCP-1 gradient and fibrocyte accumulation	Using NET markers to assess inflammation, fibrocyte accumulation, and scar formation	[117]
STEMI patients	High levels of NET in thrombi linked to cardiac deaths and stent thrombosis	Targeting NET to reduce cardiac deaths and stent thrombosis in STEMI patients	[118]
STEMI patients	NET associated with systemic inflammation and less advanced atherosclerosis	Targeting NET and systemic inflammation to reduce adverse cardiovascular outcomes	[119]

## Data Availability

Data sharing not applicable to this article as no datasets were generated or analysed during the current study.
